# Genetic Augmentation of Legume Crops Using Genomic Resources and Genotyping Platforms for Nutritional Food Security

**DOI:** 10.3390/plants11141866

**Published:** 2022-07-18

**Authors:** Romesh K. Salgotra, Charles Neal Stewart

**Affiliations:** 1 School of Biotechnology, Sher-e-Kashmir University of Agricultural Sciences & Technology of Jammu, Chatha, Jammu 190008, India; 2 Department of Plant Sciences, University of Tennessee, Knoxville, TN 37996, USA

**Keywords:** genetic augmentation, leguminous crops, genomic resources, genotyping platforms

## Abstract

Recent advances in next generation sequencing (NGS) technologies have led the surge of genomic resources for the improvement legume crops. Advances in high throughput genotyping (HTG) and high throughput phenotyping (HTP) enable legume breeders to improve legume crops more precisely and efficiently. Now, the legume breeder can reshuffle the natural gene combinations of their choice to enhance the genetic potential of crops. These genomic resources are efficiently deployed through molecular breeding approaches for genetic augmentation of important legume crops, such as chickpea, cowpea, pigeonpea, groundnut, common bean, lentil, pea, as well as other underutilized legume crops. In the future, advances in NGS, HTG, and HTP technologies will help in the identification and assembly of superior haplotypes to tailor the legume crop varieties through haplotype-based breeding. This review article focuses on the recent development of genomic resource databases and their deployment in legume molecular breeding programmes to secure global food security.

## 1. Introduction

To feed the ever-growing population, the productivity of legume crops should be enhanced with available limited natural resources. Narrow genetic variation can lead to crop losses from pests, as well as provide suboptimal grain quality. Legumes are mostly used complementary to cereals in human diet globally. The dry seeds of legumes (known as pulses) are rich in dietary proteins [1,2]. Dry legumes are also the good sources of carbohydrates, minerals, fibre, and vitamins, and they help in the alleviation of nutrient deficiencies of undernourished populations [3]. In the sustainable development scenario that also includes climate change, legumes will be a prime income source of most of the developing and under-developing countries [4]. However, to feed the ever-increasing world population of 9.7 billion by 2050 [5], the agriculture production, including legumes, has to be increased by 70% [6,7,8,9].

Although legume crops provide nutritional food security, improvement in these crops has lagged behind the major cereal crops because of low investments and poor agricultural policy decisions [10]. In addition, genetic resources of legumes have not been fully explored and exploited, due to the non-availability of genomic resources. However, plant breeders using conventional methods for genetic improvement has proven to be only partially successful in improving complex traits of legumes [11,12]. The rich genetic resources of legumes, such as wild relatives, landraces, and pre-breeding material, have remained largely unexploited due to limited genomic resources [13].

The genomic resources, such as genome and germplasm sequencing, sequencing-based trait mapping, molecular markers and genetic maps, as well as gene expression atlases, have accelerated legume breeding programmes [14]. The advances in genomics has enabled the breeder to transfer genes and reshuffle the naturally occurring genes in different combinations for genetic improvement of the crops. With the advent of next generation sequencing (NGS) technologies, reference genomes of important legume crops, such as chickpea (*Cicer arietinum*), common bean (*Phaseolus vulgaris*), cowpea (*Vigna unguiculata*), pigeonpea (*Cajanus cajan*), groundnut (*Arachis hypogaea*), and soybean (*Glycine max*) have been sequenced [15]. These technologies revolutionized the marker technology and led the development of numerous molecular markers, such as restriction fragment length polymorphisms (RFLP) [16], randomly amplified polymorphic DNA (RAPD) [17], simple sequence repeats (SSR) [18], cleaved amplified polymorphic sequences (CAPS) [19,20], amplified fragment length polymorphisms (AFLP) [21], single nucleotide polymorphisms (SNP) [22], and diversity array technology (DArT) markers [23]. These molecular markers are helpful in various molecular studies, such as the construction of genetic maps, indirect selection, and introgression/pyramiding of quantitative trait loci (QTLs)/gene(s) in an elite variety through molecular breeding approaches, such as marker-assisted selection (MAS), marker-assisted backcross breeding (MABC), and genomic selection (GS). Besides genome-wide association studies (GWAS), SNP arrays, transcriptomic, metagenomic, epigenomic, and gene expression data have been expediting the breeding cycles of different crops [24,25]. The improvement in marker technologies, along with genome editing and high throughput phenotyping (HTP), also helps in precision breeding [26]. In major legume crops, such as chickpea, pigeonpea, cowpea, groundnut, and common bean, various genomic resources and trait-specific mapping populations have been developed. These genomic resources have been used in the characterization of germplasm, identification of diverse genotypes, QTL mapping, and identification of novel genes/alleles and their utilization in crop improvement [27,28]. Presently, the molecular markers, such as SSR and SNP markers, have been widely used for the pyramiding of important QTLs/gene(s) in various leguminous crops [29,30,31].

In recent years, the availability of low-cost technologies, such as high throughput genotyping (HTG) and HTP, enables the identification of marker–trait association more precisely. Besides, the availability of different NGS platforms and genotyping-by-sequencing (GBS)-based GWAS technologies has led the identification of marker–trait association of complex traits [24,32]. These technologies have encouraged legume breeders to utilize more sophisticated tools for GBS and GWAS to improve the resolution of molecular maps in legume crops [33]. For QTL mapping and the identification of marker–trait associations, different types of bi-parental populations, such as recombinant inbred lines (RILs), near-isogenic lines (NILs), doubled haploids (DHs), multiparent advanced generation inter-cross (MAGIC), nested association mapping (NAM), and association mapping (AM) on wider panels, are used [34]. The advanced backcross quantitative trait loci (AB-QTL) have been efficiently used in chickpea, pigeonpea, lentil (*Lens culinaris*), and groundnut [30]. This bi-parental mapping population coupled, with GBS and GWAS, enhances the resolution for the location of novel genes/alleles/QTLs [32,35,36].

Molecular breeding approaches, such as MAS, MABC/MABB, GS, and multivariate adaptive regression splines (MARS), enable the efficient use of legume crop genetic resources, possessing valuable alleles/genes, with the application of various genomics resources and improved genotyping platforms [4]. For example, four molecular markers (ICCM0249, TAA170, GA24, and STMS11) have been transferred for the development of drought tolerance varieties of chickpea through MABC. Similarly, various disease and insect pest resistant genes have been introgressed through molecular breeding methods in crops such as common bean, pea, lentil, and cowpea [37,38,39]. In chickpea, drought tolerance genes have been successfully transferred through the MARS approach in the genetic background of chickpea variety ‘JG 11′ using SSR markers [30,40,41]. For the pyramiding of QTLs/genes, the MARS technique is mostly used, which helps for tapping and the accumulation of beneficial genes with small and additive effects [41]. Unlike MAS, MABC, and MARS methods, GS is an advanced breeding approach of MAS that predicts the breeding values of a genotype based on genotypic and phenotypic data [42]. GS has been successfully used in chickpea, in which a collection of 320 elite breeding lines have been selected as the ‘training population’ to predict genomics-estimated breeding values [43].

With the advances in HTG and HTPs technologies, plant breeders have also been enriched with a vast array of genotyping platforms, along with data analytical tools [8]. Mining of favourable alleles/haplotypes, gene cloning, identification of maker-trait associations, and GS have paved the way towards molecular legume breeding methods. Advances in genomic resources and genotyping platforms helped in the delivery of a number of improved varieties of legume crops with high yields and resistance to biotic and abiotic stresses [8]. Although different genomic resources have been generated for important legume crops, the wide applicability in crop improvement is yet to be achieved. Keeping in perspective the importance of available genomic resources and legume databases, along with genotyping platforms, the present review article summarizes the current knowledge, comprehensively, on the applicability of molecular aspects in legume breeding. Moreover, the review article also presents insights gleaned on the generation of different genomic resources, databases, genotyping platforms, and their applicability in legume crops.

## 2. Development of Genomic Resources in Legume Crops

**Figure 1:**
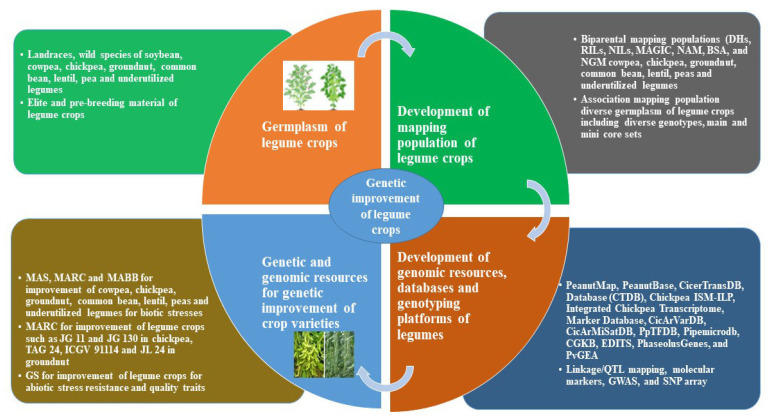
Steps involved in the development of genomic resources and deployment in legume crop improvement.

For efficient use, genetic resources, such as diverse germplasm, land races, wild relatives, and pre-breeding lines, need to be explored and developed. Recently, the improvements made in NGS, HTG, and HTP have enabled the legume breeder to develop high resolution maps and precise identification of marker–trait linkages [15,44]. However, for development of genomic resources various steps are involved such as genetic diversity analysis, crossing and development of biparental mapping populations or diverse AM population, marker–traits association and application in MARC, MABB, and GS. (Figure 1). For example, for marker–trait association studies, natural (association mapping) or bi-parental (QTL/linkage mapping) populations, such as DHs, F_2_ or F_2_ derived F_3_ population, RILs, backcrosses, NILs, NAM, and MAGIC populations, are required. For the development of bi-parental mapping populations, diverse parents are used for making crosses. However, sometimes, similar genotypes may also release genetic variation during recombination [45]. For example, bi-parental populations, such as RIL, NIL, and MAGIC, have been developed for marker–trait analysis in chickpea [46,47]. Similarly, RIL mapping populations have been developed in groundnut for the identification of marker–trait analysis [4]. MAGIC populations have been developed for the marker–trait association identification, in faba bean, for frost tolerance [48] and for heat tolerance in chickpea [49]. MAGIC populations have been developed for flowering, plant growth, seed size, and maturity traits in cowpea [50]. Similarly, to study the marker–trait association, mapping populations have been developed for seed traits in groundnut and for yield under changing climatic conditions in soybean [51,52]. Similarly, other NGS-based bulked segregant analysis (BSA) mapping populations, such as QTL-sequence (QTL-seq) and next generation mapping (NGM), have been developed for rapid gene/QTL discovery in marker–trait association [53]. Among these populations, DH, NILs, and RIL mapping populations are very stable, which can be replicated over the years and are not affected by the dominant/codominant nature of the gene(s). Following are the various approaches for the development of genomic resources and database/genotyping platforms of legume crops.

### 2.1. Linkage and QTL Mapping in Legumes

Linkage/QTL mapping has enabled the identification of the association of genomic regions with phenotypic traits, which can be further used in legume breeding approaches. For example, two QTLs were identified on linkage group LG 2 and LG 5, with 9.5–11.5% of phenotypic variance for ascochyta blight resistance in lentil [54]. Similarly, marker–trait associations have been detected between different agro-morphlogical traits, such as days to bud initiation, pod length, pods per plant, and 100 seed weight, in common bean and molecular markers [55]. In common bean, AM has been used for the identification of QTLs/gene(s) linked with iron, zinc, and protein contents [56]. Similarly, QTL mapping and the identification of marker–trait association have been carried out in faba bean for flowering time [57]. The marker–trait association has been identified for various traits in different legume crops, such as QTL for winter hardiness and leaf area in lentil [58], QTLs for number of branches and pods in soybean [59], QTL for resistance to ascochyta blight and early flowering in chickpea [60], and QTL for the determinacy gene (*Dt1*) for determinant growth habit in pigeonpea [61]. Association mapping enables the identification of functional variability between the genes of interest and phenotypic traits [62]. In association mapping, the characterization of legume-diverse germplasm, using molecular markers, helps in the development of core sets of populations for the further utilization in legume improvement programmes [63]. Moreover, the use of NGS and bioinformatics tools in the core sets enabled the development of high-resolution maps of legume crops [64]. In association mapping, linkage disequilibrium (LD) plays an important role in knowing the frequency of association of their different alleles. LD tends to remain, over many generations, with a tight linkage between the loci [8,65].

### 2.2. Genome-Wide Association Studies

GWAS plays an important role in the identification of candidate gene(s)/QTLs for complex traits. GWAS approaches have been used for detecting small and minor genetic variations associated with several biotic and abiotic stresses, as well as agronomic traits of crops [66,67]. GWAS requires genome-wide markers and scans the entire genome for detection of QTLs. GWAS has been used for the identification of candidate genes, for powdery mildew disease resistance in common bean, on chromosomes Pv04 and Pv10 [66]. Similarly, QTL for angular leaf spot resistance (*ALS11.1*) has been identified in common bean using GWAS, and, subsequently, the angular leaf spot resistance common bean varieties have been developed. The GWAS approach was also carried out for the identification of two QTLs that demonstrated the resistance against the anthracnose and angular leaf spot diseases of common bean [68].

Different QTLs were also identified for various abiotic stress tolerant genes through the GWAS technique. The GWAS was for the identification of candidate genes for root traits associated with aluminium toxicity in common bean [69]. In the study, a significant association of SNPs was detected between the root traits of common bean and the toxic compound extrusion gene and aluminium, which activated the malate transporter gene for tolerance to aluminium toxicity. Similarly, in another GWAS, the GBS technique was used for the detection of QTLs for iron content in seeds of lentil [70]. GWAS has also been carried out in *M. truncatula* for the identification of genes regulating various seed traits under heat stress conditions [71]. Similarly, Kang et al. [72,73] identified biomass, drought, and salinity-related genes in *M. truncatula* by using the GWAS technique.

GWAS approaches played a significant role in the identification of several putative loci/genes in legume crops, such as maturation in mungbean [74], pod length in cowpea [75], seed protein and oil content in soybean [76], flowering and cooking time in common bean [77,78], seed weight in soybean [79], as well as days to first flower, days to maturity, seeds per pod, and seed weight in lentil [80]. Similarly, GWAS was carried out to identify the association of genes/alleles linked with bean fly resistance traits, such as fly damage severity, pupa count, and plant mortality rate in common bean [81]. They also detected significant variation in SNPs and the agro-morphological traits, such as days to flowering, days to maturity, pods per plant, number of seeds per pod, and grain yield in common bean [81]. Different traits linked to molecular markers have been identified through GWAS and have been deployed in legume breeding programmes [31].

### 2.3. Databases and Genotyping Platforms

Databases of different crops play an important role in comparative studies, allele mining, evolutionary and phylogenetic studies, genetic diversity, development of markers, molecular breeding, and functional genomics studies. Now, legume crops are enriched with various databases such as PeanutMap, PeanutBase [82], CicerTransDB, Database (CTDB), Chickpea ISM-ILP, Integrated Chickpea Transcriptome, Marker Database, CicArVarDB, CicArMiSatDB [83,84,85], PpTFDB, Pipemicrodb [86], CGKB, EDITS [87], PhaseolusGenes, and PvGEA [88]. These databases also help in the development of various genomic resources of legume crop species to be used in molecular breeding programmes [13].

With the advances in NGS technologies, different genotyping platforms and assays have been developed. These platforms are available in different ranges, such as 1–10 SNPs (low density), 2–10 K SNPs (medium-density), and more than 20K SNPs (high-density) [8]. In addition, the availability of reference genomes of legumes, along with HTG and HTP techniques, pave the way for the identification of close association between the phenotypic trait and the gene of interest. Evolutionary studies using SNPs require a genotyping platform or assay developed for different legume crops, such as AxiomCajanus SNP array with 56K SNPs for pigeonpea crop [13], Affymetrix Axiom with 58 K for groundnut [89], Affymetrix Axiom with 50 K for chickpea [90], Illumina Infinium with 51 K for cowpea [91], Illumina Goldengate assay with 768 K for common bean [92], and Illumina Infinium with 6 K for soybean [93] (Table 1). The genotyping platforms are mostly based on SNP markers, and they are substantial when being used for background selection in molecular breeding methods.

## 3. Deployment of Genomic Resources and Genotyping Platforms in Legume Breeding

The downstream deployments of genomic resources and genotyping platforms enabled breeders to introgress/pyramid genes of interest with more precision and faster. Genomic resources have made remarkable changes in legume crops after the introgression/pyramiding of gene(s)/QTLs through molecular breeding approaches. Numerous studies have shown the success of genomic and genotyping platform resources in the improvement of yield, quality traits, and combating biotic and abiotic stresses in legume crops [115,116]. These approaches helped the genetic augmentation of various traits of legume crops [117,118,119,120]. Though substantial genetic gain has been achieved through MAS/MABC, the issues related with minor genes/QTLs yet needs to be addressed, particularly in minor and underutilized legumes. Moreover, the minor genes/QTLs genetic variations are related to complex traits, which remained unexplored in legume crops [121]. To exploit these genetic variations, an improvement has been made, over MAS, for the selection of desirable genotypes, with high breeding values on the basis of genome-wide marker information [122]. These approaches helped in the improvement of various traits of legume crops such as rust and ascochyta resistance in lentil, pod borer and Phytopthora stem blight in pigeonpea, as well as root rot in pea [117,118,119,120]. GS has proved to be the most suitable approach for the improvement of complex traits with additive effects [123]. GS estimates the additive effect of genome wide markers to estimate the effects of all loci and, thereby, compute the GEBV for a reliable and comprehensive selection [124,125]. GS captured the alleles/QTLs, having both small and low heritable effects, controlling the traits [126,127]. For the improvement of complex traits, many breeders have deployed the GS approach in legume breeding programmes [128]. Following are the important legume crops in which genomic resources and genotyping platforms have been developed, which are efficiently used for introgression/pyramiding of gene(s)/QTLs through MABC/MABB and GS approaches (Table 2).

### 3.1. Cowpea (Vigna unguuiculata)

Cowpea is an important grain legume crop; however, the production is limited by several biotic and abiotic stresses. Being a self-pollinating crop, a number of mapping populations, such as RILs, NILs, and MAGIC, have been developed by crossing the cultivated genotypes of cowpea with its wild relatives [157,158,159,160]. The bi-parental mapping population was developed for QTLs identification of floral scent compounds in cowpea [161]. Huynh et al. [50] developed a MAGIC mapping population for QTLs mapping for traits such as flowering, plant growth, seed size, and maturity in cowpea. Similarly, a MAGIC mapping population has been developed for the detection of SNP markers associated with salt tolerance in cowpea [162]. GWAS was used for the detection of several putative loci/genes for pod length and flowering time traits in cowpea [75,163]. Similarly, in GWAS, GBS was used for the identification of SNPs linked to grain, fodder, and pod types in cowpea [164]. The developed genomic resources have facilitated the transfer of genes/QTLs, using molecular breeding methods, into well adapted varieties [91]. Due to the development of SNP-based genotyping platforms, these platforms have been used in MAS, MABC, and GS methods more efficiently, particularly in background selections [129]. For example, the mosaic virus (CpMV) resistant in cowpea has been introgressed by using the MABC approach [38]. Similarly, MABC and GS methods were used for the introgression of drought tolerance in cowpea [129,162].

### 3.2. Soybean (Glycine max)

Soybean has health benefits due to the presence of tocopherols and isoflavones, in addition to high protein and oil contents. Two major QTLs (*qpn-Chr11* and *qpn-Chr20*) were detected for pod number in soybean [165]. QTLs for yield and its related traits have been identified in a MAGIC population of cowpea under different climatic conditions [51,52]. Similarly, QTLs/gene(s) were identified in different mapping populations of soybean associated with number of branches and pods [59], resistance to leaf rust [142], brown stem rot resistance genes (*Rbs1* and *Rbs2*) [143], and resistance to stain frogeye [144]. Hwang et al. [76] used GWAS to identify SNPs linked to seed weight, seed protein, and oil content in soybean [79]. The identified gene(s)/QTLs have been successfully transferred into the soybean cultivars through molecular breeding methods. Cyst nematode resistant genotypes of soybean have been developed by stacking resistance alleles, from wild to the cultivated varieties of soybean, using MAS and MARC methods [141]. Ramalingam et al. [145] improved the host-plant resistance to *Phytophthora* rot and powdery mildew in soybean by the introgression of resistance genes *Rps2* (*Phytophthora* rot resistance) and *Rmd-c* (powdery mildew resistance), along with a gene (*rj2*) linked with nodulation. Three soybean mosaic virus resistance genes—namely, *RSC4*, *RSC8*, and *RSC14Q*—have been pyramided in soybean through the MABC programme [146]. Kumar et al. [147,148] developed a Kunitz trypsin inhibitor (KTI)-free soybean variety through MABC programmes. Similarly, a null allele of KTI-free soybean has been introgressed in soybean by using MABC [149]. In soybean, the off-flavour generating lipoxygenase-2 gene has been eliminated from the seed through the MABC approach [150]. The GS approach was used for the genetic enhancement of the soybean crop for various traits, such as maturity, plant height, seed weight, and grain yield [159].

### 3.3. Chickpea (Cicer arietinum)

Cultivated chickpea has been domesticated from the wild progenitor, *C. reticulatum* Ladiz. [166]. The small-seeded *desi* and the large-seeded *kabuli* are the two main varieties cultivated worldwide [167]. The large-seeded kabuli type chickpea originated from the small-seeded *desi* type chickpea varieties through artificial selection by the legume breeders [168]. The draft genome sequence of chickpea (kabuli variety) was published in 2013, along with the re-sequencing of 29 elite diverse chickpea varieties (17 *desi* and 12 *kabuli*), for elucidating genetic relationships and diversity among the chickpea accessions [24]. However, in the same year, the draft genome sequence of *desi* chickpea variety—namely, ICC4958—was also published [169], which was later released in 2015. Wild relatives of chickpea have been used for the development of various mapping populations, such as RILs, NILs, and BC, for the identification of marker–trait relationships for traits such as 100-seed weight, flowering time, plant hairiness, number of branches per plant, pod number, and seed yield per plant [82,170,171,172]. Bajaj et al. [170], in a GWAS comprising of cultivated (*desi* and *kabuli*) and wild chickpea species, have identified 15 SNPs associated with the seed coat colour of chickpea. In addition, GS models have been applied in chickpea crops, by genotyping 320 breeding lines with 3000 DArT-Seq markers, to estimate the breeding values of 100 grain weight and seed yield per plant traits [90]. Several traits of legume crops have been improved using MABC/MABB approaches, such as fusarium wilt resistance in chickpea [24], root traits in varieties JG 11 and JG 130 [24,30], and ascochyta blight resistance in variety C 214 [156].

### 3.4. Common Bean (Phaseolus vulgaris)

Common bean is a highly nutritious crop and is enriched with proteins, vitamins, minerals, and fibre [173]. Common bean legume is cultivated with both bush and climbing types of growth habits [174]. A reference genome, 473 Mb, of the 587-Mb genome of common bean has been assembled in 11 chromosome-scale pseudomolecules for an inbred landrace (G19833) derived from the Andean pool [175]. The genome of the Mesoamerican common bean genotype BAT93, encompassing 549.6 Mb with 81% of the assembly, was anchored to 11 linkage groups [175]. Whole-genome sequencing of 37 varieties belonging to *P. vulgaris*, *P. acutifolius*, and *P. coccineus* L. revealed a large number of inter-gene pool introgressions and enabled the mapping of interspecific introgressions for disease resistance in breeding lines of common bean [176]. In common bean, bi-parental populations, such as RILs, NILs, and backcrosses, have been developed for the identification of QTLs/gene(s) linked with traits, such as days to flowering, plant height, seed size, seed weight, seed size, yield, plant height, and concentration of minerals such as iron and zinc in common bean seeds [177]. An association mapping study of 683 landraces and breeding lines has been carried out for the identification of QTLs associated with traits such as flowering time, seed size, and harvest maturity traits [176]. The identified QTLs/genes have been successfully introgressed into cultivated varieties of common bean using molecular breeding methods. Miklas and Kelly [178] introgressed the *Co-42* resistance gene into ‘Pinto bean’ variety through MAS to combat the emerging anthracnose disease problem in North Dakota. The sclerotinia white mould resistance genes have been transferred from the common bean resistant variety ‘G122′ into a susceptible variety ‘Pinto bean’ using Phs SCAR marker through MABC approach [179]. MABC approach has been used for transferring the anthracnose resistance genes into Andean climbing beans [134]. Obala et al. [180] pyramided fusarium root rot resistance genes in the common bean variety through the MABC method. Nzungize, et al. [181] introgressed the pythium root rot resistance gene into ‘Rwandan’ susceptible common bean cultivars through MAS. Anthracnose resistant varieties have been developed by the introgression of resistant genes using SCAR-markers, such as SAB3 and SBB14, through the MABC programme [182]. Similarly, Diaz et al. [132] introgressed the drought tolerance trait in the common bean through MABC approach. The genomic selection approach was used for the characterization of a dataset of 481 genotypes with 5820 SNP markers for the prediction of traits, such as days to maturity and grain yield in common bean [183].

### 3.5. Groundnut (Arachis hypogaea)

Groundnut (peanut), one of the most important and nutritious leguminous crops, which is also known as poor man’s almonds because of its high nutritional content with protein and fat. The genomes of both the diploid progenitors of groundnut, i.e., *Arachis duranensis* PI475845 (A genome) and *Arachis ipaensis* ICG 8206 (B genome), have been sequenced. This has led to the development of a large number of genomic resources to be used in groundnut breeding programmes [184]. In groundnut, bi-parental populations, such as RIL and MAGIC populations, have been developed for the identification of association between molecular markers and different seed traits in groundnut [4,25]. Three elite groundnut varieties resistant to rust disease have been developed using MARC [151]. Chu et al. [153] pyramided *Rma*, *ahFAD2A*, and *ahFAD2B* genes for resistance to nematodes, along with high oleic acid in a groundnut variety, ‘Tifguard’, through the MABC method. Janila et al. [154] has introgressed foliar fungal disease resistant genes in the background of three groundnut varieties: namely, TAG 24, ICGV 91114, and JL 24. The introgressed varieties showed 39–79% higher mean pod yield and haulm yield, along with resistance to diseases. Similarly, the resistance genes for foliar disease resistance and high oleic acid have been introgressed into three popular Indian groundnut varieties, such as GJG 9, GG 20, and GJGHPS 1, through MABC methods in which background selection was carried out using a groundnut SNP array [155]. The GS approach was carried out in 281 genotypes of groundnut for genetic the improvement of traits such as days to 50% flowering, leaflet length, days to maturity, 100 grain weight, seed dimension traits, and seed dimension [185].

### 3.6. Pigeonpea (Cajanus cajan)

Pigeonpea is an important legume crop, and the demand for pigeonpea, as a pulse crop, is increasing worldwide [186]. There is no doubt that the cultivation area of pigeonpea is increasing, particularly in developing and under developed countries. However, yield has stagnated due to various biotic and abiotic stresses. Little improvement has been made for the last three and a half decades because of the non-availability genomic resources and the narrow genetic base in pigeonpea genotypes. However, the advances in NGS and high throughput technologies has led the development of legume crop genomic resources, such as high-density maps, molecular markers, and analytical tools [4]. Recently, pigeonpea has been enriched in various genomic resources and gene(s)/QTLs associated with morphological, quality, and biotic and abiotic stresses [187,188]. Different bi-parental populations, such as RILs and backcross/segregating populations, have been developed for the mapping of gene(s)/QTLs for various traits of pigeonpea, such as fusarium wilt, fertility restoration, determinacy and sterility mosaic disease, and other important agronomically traits [119]. The identified QTLs/gene(s), such as pod borer and *Phytopthora* stem blight resistance genes, have been successfully introgressed into the cultivated varieties of pigeonpea, along with the yield traits through MAS [119]. MABC was used for introgressing sterility mosaic disease and fusarium wilt-resistance genes in pigeonpea varieties LRG41 and LRG52. The improved varieties of pigeonpea exhibited complete resistance against both the diseases. MABC approaches have also been used for the improvement of other pigeonpea varieties, such as BDN 711, ICP 8863, TS 3R, JKM 188, TGT 501, and UPAS 120 [187].

### 3.7. Lentil (Lens culinaris)

Lentil is an important rainfed and cool season pulse crop, having a genome size of 4 Gbp [100]. Its high protein content (22–35%) makes it one of the most nutritionally-rich pulse crops. It also provides fibre, minerals, and carbohydrates. Earlier limited genomic resources were available for improvement of lentil crop compared to chickpea, common bean, soybean and pigeonpea crops due to a narrow genetic base, large genome size, and low density genetic maps [33]. However, with the advances in NGS and HTP techniques, various genomic resources of lentil crop are now available, which can be efficiently used for lentil improvement through MAS/MABS and GS programmes [28]. Different RIL and backcross populations developed by crossing the cultivated and wild species of lentil for tagging of gene(s)/QTLs linked to traits such as drought, cold, earliness, rust resistance, fusarium wilt resistance, increased iron and zinc content [28,148]. Crosses have been attempted between cultivated and wild relatives of lentil for identification of marker–trait association for complex traits such as maturity, seed yield, anthracnose resistance, biomass, seed weight, straw yield, podding ability, ascochyta blight, and harvest index [189,190]. In addition, the application of NGS techniques in various mapping populations exhibited an association of SNPs with complex traits [191]. In lentil, traits such as pod indehiscence, early flowering, anthocyanin in stem, seed coat pattern, radiation frost tolerance locus, ground colour of the seed, and flower colour, linked to SSR and SNPs, have been transferred through MAS and MABC programmes [189,192,193,194].

### 3.8. Pea (Pisum sativum)

Pea is the third most important crop in the world and is a major source of protein in the human diet [195]. In pea crop, various types of mapping populations (backcross, AB-QTL, and RIL) have been developed for the identification of gene(s)/QTLs linked to phenotypic traits, such as QTLs for resistance to white mould [196], QTL for resistance to *Mycosphaerella pinodes* [197], QTL for salt tolerance in pea [140], as well as *er1*, *er2*, and *Er3* genes resistance to powdery mildew in pea [138,139,198]. Novel SNPs have been identified by using GBS in RIL mapping populations of pea [195], and the identified genes/QTLs have been successfully introgressed into the elite varieties for further improvement through molecular breeding methods. The MABC approach was used for the introgression of genes linked to SSR markers for partial resistance to *Aphanomyces* root rot in pea. In the study, genotyping platform 13.2K SNP array was used in background selection of pea crop [120]. Likewise, 339 genotypes were genotyped using GS with a 13.2K SNP array genotyping platform for the improvement of traits such as days to flowering and 1000-seed weight [137]. Similarly, GS was carried out in 306 pea RILs for flowering initiation, seed weight, improving grain yield, winter plant survival, and lodging susceptibility [199].

### 3.9. Underutilized Legume Crops

Crops such as chickpea, cowpea, pigeonpea, groundnut, common bean, lentil, and pea benefit from a relatively mature research base and genomic resource foundation [9]. However, very limited genomic resources are available in underutilized legume crops, such as faba bean (*Vicia faba*), urdbean (black gram) (*Vigna mungo*), green gram (mungbean) (*Vigna radiate*), adzuki bean (*Vigna angularis*), *lobia* (*Vigna unguiculata* subsp. *unguiculata*), bambara groundnut (*Vigna subterranean*), moth bean (*Vigna aconitifolia*), and rice bean (*Vigna umbelatta*). The production and productivity of underutilized crops are low, partially, because of the lack of proper management practices and use of negligible inputs; however, these crops possess high nutritional values [200,201]. Nonetheless, these legume crops are resilient to various biotic and abiotic stresses, and they possess improved grain and fodder quality traits. These crops play an important role for future food security, keeping in view the impact of climate change. Moreover, the underutilized leguminous crops are enriched with a plethora of valuable gene(s), which can be mapped and cloned for further utilization in crop improvement [202]. Keeping in view the economic importance of underutilized crops, these crops need to be enriched with genomic resources for further improvement. However, few molecular studies have been conducted for the identification of candidate gene(s)/QTLs linked with the traits, such as plant height, flower initiation, maturity, and yield traits in narrow-leafed lupin [203]. In a GWAS, four candidate genes associated with flower initiation (*Lup019134, Lup015264, Lup021911*, and *Lup021909*), two genes with maturity (*Lup015264* and *Lup004734*), one gene with plant height (*Medtr1g030750*), and two genes with yield traits (*Lup021835* and *Lup022535*) were identified in lupin [203]. Similarly, SNPs linked with phosphorus utilization and phosphorus uptake efficiency traits were identified in 120 mungbean genotypes by using the GBS technique in a GWAS approach [204]. In that study, six candidate genes, such as *VRADI09G09030*, *VRADI05G20860*, *RADI01G04370*, *VRADI08G20910*, *VRADI06G12490*, and *VRADI08G00070*, exhibited linkage with phosphorus utilization and phosphorus uptake efficiency have been identified [204]. In lupin, the PCR-based molecular marker for *LanFTc1* was found to be associated with vernalization responsiveness [205]. Likewise, a low alkaloid content gene (*LAGI01_35805_F1_R1*), linked to the *pauper* locus in lupin, was identified that has been transferred through MAS for development of low-alkaloid genotypes [205]. Similarly, MAS was used for the introgression of bruchid resistance in mothbean linked with DMB-SSR160 and CEDG261 molecular markers [206]. These underutilized legume crops will be further improved if HTG and HTP technologies are to be deployed for the development of different genomic resources.

## 4. Conclusions and Future Perspectives

Earlier, limited genomic resources were available for the genetic enhancement of legume crops compared to main rice, maize, wheat, and other grains. Now, the legume breeder can enhance the genetic potential of a variety by reshuffling native genes in different combinations using various genomic resources. Genomic resources, along with genotyping platforms, play significant roles in the improvement of crop varieties and different types of genetic stock; these must continue to be developed for use in future legume breeding programmes. Although molecular breeding methods have been efficiently used in chickpea, groundnut, pigeonpea, common bean, and cowpea, there is wide scope for the development of genomic resources in underutilized legume crops for further improvement. There is a need to develop various bi-parental mapping populations for the identification of marker–trait association in underutilized crops such as faba bean, urdbean, green gram, adzuki bean, *lobia*, bambara groundnut, moth bean, and rice bean.

The advances in HTG and HTP techniques are helpful in understanding genome structure, function, and identification of marker–trait relationships in legumes. These genomic tools also enable breeders to identify allelic variation caused by a number of small effects of genes/QTLs of complex traits in legumes. The advances in genomic resources will further help in purging out the deleterious loci in the variety and accumulation of important alleles for designing future crop varieties. Furthermore, for accelerating the adoption of molecular techniques in legume breeding programmes, molecular markers should be ‘breeder friendly markers’ that are highly reproducible, easily assayable, and are relatively inexpensive for genotyping. For example, SNPs are known as the choice of the molecular markers, and conversion of identified SNPs to cleaved amplified polymorphic sequences (CAPs) or KASPar assays and Illumina Veracode will enable their wider application in legume crop improvement programmes. There is also a need to capture and identify the allelic variations across the genome sequences of different legume species (pangenome) and even across genus (super-pangenome). In the future, haplotype-based breeding will help in the identification of superior haplotypes in leguminous crops, which can be deployed in legume breeding programmes.

## Figures and Tables

**Table 1:** Availability of genomic resources of important legume crops.

Crop		Genomic Resources	References
Groundnut (*Arachis hypogaea*)	Species	*Arachis hypogaea* (Tetraploid) *Arachis duranensis* and *Arachis ipaensis* (Diploid)	[94]
	Genome size	2890 Mbp (Tetraploid) 1260 Mbp (Diploid)	[95]
	Genetic maps	Diploid (AA)-3, Diploid (BB)-2, Tetraploid-13 maps, and one reference consensus map	[29]
	BAC libraries	ca. 5.3×–Diploid (BB); ca. 7.4×–diploid (AA)	[96]
	DArT clones	ca. 15,000	[97,98]
	SNPs array	2000 SNPs, 58 K Affymetrix Axiom	[29,89]
	TILLING population	3400 mutant M2 lines	[99]
	Database	PeanutMap, PeanutBase	[82]
Chickpea (*Cicer arietinum*)	Species	Diploid	[100]
	Genome size	740 Mbp	[95]
	Genetic maps	24 (15 inter-specific & 9 intra-specific)	[101,102,103]
	BAC libraries	10×	[14]
	DArT clones	5397	[14]
	SNPs array	>9000 50 K Affymetrix Axiom	[90]
	InDel markers	231,658 InDels	[104]
	Physical maps	BAC/BIBAC-based, BAC-based	[24]
	Database	CicerTransDB, Chickpea ISM-ILP, Marker Database, Integrated Chickpea Transcriptome, Database (CTDB), CicArVarDB, CicArMiSatDB	[83,84,85]
	Number of genes	28,269	[24]
	Number of ESTs	46,064	[24]
Pigeonpea (*Cajanus cajan*)	Species	Diploid	[105]
	Genome size	833.07 Mbp	[95]
	Genetic maps	Reference genetic map, six intra-specific maps, one consensus map and DArT based maternal and paternal maps	[106,107]
	BAC libraries	11×	[1]
	DArT clones	15,360	[99,107]
	SNPs array	>10,000 50 K Affymetrix Axiom	[13]
	TILLING population	ca.5000 mutant lines	[99]
	Database	PpTFDB, Pipemicrodb	[86]
	Number of genes	48,680	[105]
	Number of ESTs	25,640	[105]
Cowpea (*Vigna unguiculata*)	Species	Diploid	[100]
	Genome size	613 Mbp	[95]
	Database	CGKB, EDITS	[87]
	SNP array	51 K Illumina Infinium	[91]
Common bean (*Phaseolus vulgaris*)	Species	Diploid	[108]
	Genome size	578 Mbp	[95]
	Database	PhaseolusGenes, PvGEA	[109]
	SNP array	768 K Illumina Goldengate assay 6 K Illumina Infinium BeadChip	[92,110]
Soybean (*Glycine max*)	Species	Diploid	[111]
	Genome size	950 Mbp	[95]
	Database	SoyBase, SoyGD	[88]
	SNP array	50 K Illumina Infinium BeadChip 6 K, Illumina Infinium 180 K Affymetrix Axiom 355 K Affymetrix Axiom	[93,112,113,114]

**Table 2:** Genomic resources and genotyping platforms for the improvement of legume crops.

Crop	Molecular Breeding Approaches	Trait(s) Improved	Reference
Cowpea	MABC	Mosaic virus (CpMV) resistant	[38]
	MABC	Root-knot nematode Resistance	[129]
	Cowpea KASP genotyping platform	Background selection	[129]
Common bean	QTL mapping (RIL population)	Improved drought adaptation	[130,131,132]
	Fine-mapping	Resistance against angular leaf spot	[37,39]
	Meta-QTL	Resistance against white mold	[133]
	MABC	Anthracnose resistance	[134]
	QTL mapping	Bruchid and virus resistance	[135]
Lentil	QTL mapping	Ascochyta blight resistance	[118]
	QTL mapping	Rust resistance	[117]
	QTL mapping	Salt tolerance	[136]
Pea	QTL mapping with 13.2 K SNP array	Resistance against Aphanomyces root rot	[120]
	13.2K SNP array	days to flowering and 1000-seed weight	[137]
	QTL mapping	Resistance against powdery mildew	[138,139]
	QTL mapping	Salt tolerance	[140]
Soybean	MAS and MABC	Several soybean cyst nematodes and multiple disease-resistant genotypes	[141]
	QTL mapping	Resistance to leaf rust	[142]
	QTL mapping	Black pod-of-staff	[143]
	QTL mapping	Resistance to stain frogeye	[144]
	MABB	Powdery mildew diseases resistance	[145]
	MABC	Soybean mosaic virus (SMV) resistance	[146]
	MABC	Free kunitz trypsin inhibitor	[147,148,149]
	MABC	Eliminate lipoxygenase-2,	[150]
Groundnut	MABC	Introgression lines showing higher yield and increased rust resistance	[151]
	MABC	Resistance to nematode	[152,153]
	MABC SSR markers and SNP array	Enhanced oleic acid	[154,155]
Chickpea	MABC	Resistance to fusarium wilt	[24,156]
	MABC	Resistance to ascochyta blight	[156]
	MABC	Drought tolerance	[40]
	MABC	Eliminate lipoxygenase-2,	[150]
	3000 DArT-Seq markers	Breeding values of traits 100 grain weight and seed yield per plant	[90]

## Data Availability

All data is available within the manuscript.

## References

[1] Bohra A., Dubey A., Saxena R.K., Penmetsa R.V., Poornima K.N., Kumar N., Farmer A.D., Srivani G., Upadhyaya H.D., Gothalwal R. (2011). Analysis of BAC-end sequences (BESs) and development of BES-SSR markers for genetic mapping and hybrid purity assessment in pigeonpea (*Cajanus* spp.). BMC Plant Biol..

[2] Varshney R.K., Kudapa H., Pazhamala L., Chitikineni A., Thudi M., Bohra A., Gaur P.M., Janila P., Fikre A., Kimurto P. (2015). Translational genomics in agriculture: Some examples in grain legumes. Crit. Rev. iPlant Sci..

[3] Kumar V., Khan A.W., Saxena R.K., Garg V., Varshney R.K. (2016). First generation HapMap in *Cajanus* spp. reveals untapped variations in parental lines of mapping populations. Plant Biotechnol. J..

[4] Varshney R.K., Pandey M.K., Bohra A., Singh V.K., Thudi M. (2018). Toward the sequence-based breeding in legumes in the post-genome sequencing era. Theor. Appl. Genet..

[5] UNO (2019). United Nations Organization, Department of Economic and Social Affairs, Population Division. World Population Prospects 2019: Highlights. https://population.un.org/wpp/publications/files/wpp2019_highlights.pdf.

[6] Godfray H.C.J., Beddington J.R., Crute I.R., Haddad L., Lawrence D., Muir J.F. (2010). Food security: The challenge of feeding 9 billion people. Science.

[7] Massawe F., Mayes S., Cheng A. (2016). Crop diversity: An unexploited treasure trove for food security. Trends Plant Sci..

[8] Varshney R.K., Bohra A., Yu J., Graner A., Zhang Q., Sorrells M.E. (2021). Designing future crops: Genomics-assisted breeding comes of age. Trends Plant Sci..

[9] Salgotra R.K., Sood M., Jasrotia M., Salgotra R.K., Sood M., Jasrotia M. (2021). Underutilized Crops and Their Value Addition.

[10] Foyer C.H., Lam H.M., Nguyen H.T., Siddique K.H.M., Varshney R.K., Colmer T.D., Cowling W., Bramley H., Mori T.A., Hodgson J.M. (2016). Neglecting legumes has compromised human health and sustainable food production. Nat. Plants.

[11] Mikic A., Peric V., Dordevic V., Srebric M., Mihailovic V. (2009). Anti-nutritional factors in some grain legumes. Biotechnol. Anim. Husb..

[12] Gnanasambandam A., Paull J., Torres A., Kaur S., Leonforte T., Li H., Materne M. (2012). Impact of molecular technologies on faba bean (*Vicia faba* L.) breeding strategies. Agronomy.

[13] Saxena R.K., Rathore A., Bohra A., Yadav P., Das R.R., Khan A.W., Singh V.K., Chitikineni A., Singh I.P., Kumar C.V.S. (2018). Development and application of high-density Axiom-Cajanus SNP array with 56 K SNPs to understand the genome architecture of released cultivars and founder genotypes. Plant Genome.

[14] Thudi M., Bohra A., Nayak S.N., Varghese N., Shah T.M., Penmetsa R.V., Thirunavukkarasu N., Gudipati S., Gaur P.M., Kulwal P.L. (2011). Novel SSR markers from BAC-end sequences, DArT arrays and a comprehensive genetic map with 1291 marker loci for chickpea (*Cicer arietinum* L.). PLoS ONE.

[15] Afzal M., Alghamdi S.S., Nawaz H., Migdadi H.H., Altaf M., El-Harty E., Al-Fifi S.A., Sohaib M. (2022). Genome-wide identification and expression analysis of CC-NB-ARC-LRR (NB-ARC) disease-resistant family members from soybean (*Glycine max* L.) reveal their response to biotic stress. J. King Saud Univ..

[16] Botstein D., White R.L., Skolnick M., Davis R.W. (1980). Construction of a genetic linkage map in man using restriction fragment length polymorphism. Am. J. Hum. Genet..

[17] Williams J., Kubelik A., Livak K., Rafalski J., Tingey S. (1990). DNA polymorphisms amplified by arbitrary primers are useful as genetic markers. Nucleic Acids Res..

[18] Tautz D. (1989). Hypervariability of simple sequences as a general source of polymorphic DNA markers. Nucleic Acids Res..

[19] Konieczny A., Ausubel F.M. (1993). A procedure for mapping Arabidopsis mutations using co-dominant ecotype-specific PCR-based markers. Plant J..

[20] Paran I., Michelmore R.W. (1993). Development of reliable PCR-based markers linked to downy mildew resistance genes in lettuce. Theor. Appl. Genet..

[21] Vos P., Hogers R., Bleeker M., Reijans M., van de Lee T., Hornes M., Frijters A., Pot J., Peleman J., Kuiper M. (1995). AFLP: A new technique for DNA fingerprinting. Nucleic Acids Res..

[22] Gupta P.K., Roy J.K., Prasad M. (2001). Single nucleotide polymorphisms: A new paradigm for molecular marker technology and DNA polymorphism detection with emphasis on their use in plants. Curr. Sci..

[23] Jaccoud D., Peng K., Feinstein D., Kilian A. (2001). Diversity arrays: A solid state technology for sequence information independent genotyping. Nucleic Acids Res..

[24] Varshney R.K., Mohan S.M., Gaur P.M., Gangarao N.V.P.R., Pandey M.K., Bohra A., Sawargaonkar S.L., Chitikineni A., Kimurto P.K., Janila P. (2013). Achievements and prospects of genomics-assisted breeding in three legume crops of the semi-arid tropics. Biotechnol. Adv..

[25] Pandey M.K., Roorkiwal M., Singh V., Ramalingam A., Kudapa H., Thudi M., Chitikineni A., Rathore A., Varshney R.K. (2016). Emerging genomic tools for legume breeding: Current status and future prospects. Front. Plant Sci..

[26] Salgotra R.K., Stewart C.N. (2020). Functional markers for precision plant breeding. Int. J. Mol. Sci..

[27] Salgotra R.K., Thompson M., Chauhan B.S. (2021). Unravelling the genetic potential of untapped crop wild genetic resources for crop improvement. Conser. Genet. Resour..

[28] Kumar J., Sen Gupta D., Baum M., Varshney R.K., Kumar S. (2021). Genomics-assisted lentil breeding: Current status and future strategies. Legume Sci..

[29] Pandey M.K., Monyo E., Ozias-Akins P., Liang X., Guimaraes P., Nigam S.N., Upadhyaya H.D., Janila P., Zhang X., Guo B. (2012). Advances in Arachis genomics for peanut improvement. Biotechnol. Adv..

[30] Thudi M., Gaur P.M., Krishnamurthy L., Mir R.R., Kudapa H. (2014). Genomics-assisted breeding for drought tolerance in chickpea. Funct. Plant Biol..

[31] Varshney R.K. (2016). Exciting journey of 10 years from genomes to fields and markets: Some success stories of genomics- assisted breeding in chickpea, pigeonpea and groundnut. Plant Sci..

[32] Varshney R.K., Terauchi R., McCouch S.R. (2014). Harvesting the promising fruits of genomics: Applying genome sequencing technologies to crop breeding. PLoS Biol..

[33] Kumar J., Gupta D.S. (2020). Prospects of next generation sequencing in lentil breeding. Mol. Biol. Rep..

[34] Bauchet G.J., Bett K.E., Cameron C.T., Campbell J.D., Cannon E.K., Cannon S.B., Carlson J.W., Chan A., Cleary A., Close T.J. (2019). The future of legume genetic data resources: Challenges, opportunities, and priorities. Legume Sci..

[35] Ma Y., Qin F., Tran L.S. (2012). Contribution of genomics to gene discovery in plant abiotic stress responses. Mol. Plant.

[36] Liu S., Wang X., Wang H., Xin H., Yang X., Yan J., Li J., Tran L.S.P., Shinozaki K., Shinozaki K.Y. (2013). Genome- wide analysis of ZmDREB genes and their association with natural variation in drought tolerance at seedling stage of *Zea mays* L.. PLoS Genet..

[37] Gonçalves-Vidigal M.C., Gilio T.A.S., Valentini G., Vaz-Bisneta M., Vidigal Filho P.S., Song Q., Oblessuc P.R., Melotto M. (2020). New Andean source of resistance to anthracnose and angular leaf spot: Fine-mapping of disease-resistance genes in California Dark Red Kidney common bean cultivar. PLoS ONE.

[38] Dinesh H.B., Chandappa L., Vishwanath K.P., Singh P., Manjunatha L., Ambika D.S., Kumar M.P.K. (2018). Genetic analysis and marker assisted backcrossing for transfer of mosaic virus resistance in cowpea [*Vigna unguiculata* (L.) Walp.]. Legume Res..

[39] de Almeida C.P., de Carvalho Paulino J.F., Bonfante G.F.J., Perseguini J.M.K.C., Santos I.L., Gonçalves J.G.R., Patrício F.R.A., Taniguti C.H., Gesteira G.S., Garcia A.A.F. (2021). Angular leaf spot resistance loci associated with different plant growth stages in common bean. Front. Plant Sci..

[40] Varshney R.K., Gaur P.M., Chamarthi S.K., Krishnamurthy L., Tripathi S., Kashiwagi J., Samineni S., Singh V.K., Thudi M., Jaganathan D. (2013). Fast-track introgression of “*QTL-hotspot*” for root traits and other drought tolerance traits in JG 11, an elite and leading variety of chickpea. Plant Genome.

[41] Bernardo R., Charcosset A. (2006). Usefulness of gene information in marker-assisted recurrent selection: A simulation appraisal. Crop Sci..

[42] Zhao Y., Gowda M., Liu W., Würschum T., Maurer H.P., Longin F.H., Ranc N., Reif J.C. (2012). Accuracy of genomic selection in European maize elite breeding populations. Theor. Appl. Genet..

[43] Roorkiwal M., Rathore A., Das R.R., Singh M.K., Srinivasan S., Gaur P.M., Bharadwaj C., Tripathi S., Hickey J.M., Jannink J.L. Towards deploying genomic selection in chickpea breeding. Proceedings of the Interdrought IV Conference.

[44] Salgotra R.K., Zargar S.M. (2020). Rediscovery of Genetic and Genomic Resources for Future Food Security.

[45] Singh A.K., Prasanna B.M., Jan S., Chittaranjan K. (2016). Molecular Mapping in Crop Plants: Development and Characterization of Mapping Populations. Genetics, Genomics and Breeding of Vegetable Brassicas.

[46] Grewal R.K., Lulsdorf M., Croser J., Ochatt S., Vandenberg A., Warkentin T.D. (2009). Doubled-haploid production in chickpea (*Cicer arietinum* L.): Role of stress treatments. Plant Cell Rep..

[47] Hale B., Ferrie A.M.R., Chellamma S., Samuel J.P., Phillips G.C. (2022). Androgenesis-based doubled haploidy: Past, present, and future perspectives. Front. Plant Sci..

[48] Sallam A., Martsch R. (2015). Association mapping for frost tolerance using multi-parent advanced generation inter-cross (MAGIC) population in faba bean (*Vicia faba* L.). Genetica.

[49] Gaur P.M., Samineni S., Thudi M., Tripathi S., Sajja S.B., Jayalakshmi V., Mannur D.M., Vijayakumar A.G., Ganga Rao N.V.P.R., Ojiewo C.O. (2019). Integrated breeding approaches for improving drought and heat adaptation in chickpea (*Cicer arietinum* L.). Plant. Breed..

[50] Huynh B.L., Ehlers J.D., Huang B.E., Muñoz-Amatriaín M., Lonardi S., Santos J.R.P., Ndeve A., Batieno B.J., Boukar O., Cisse N. (2018). A multi-parent advanced generation inter-cross (MAGIC) population for genetic analysis and improvement of cowpea (*Vigna unguiculata* L. Walp.). Plant. J..

[51] Shivakumar M., Kumawat G., Gireesh C., Ramesh S.V., Husain S.M. (2018). Identification of unique characteristics of deception from facial expression. Curr. Sci..

[52] Arrones A., Vilanova S., Plazas M., Mangino G., Pascual L., José Díez M., Prohens J., Gramazio P. (2020). The dawn of the age of multi-parent MAGIC populations in plant breeding: Novel powerful next-generation resources for genetic analysis and selection of recombinant elite. Mater. Biol..

[53] Bohra A., Pandey M.K., Jha U.C., Singh B., Singh I.P., Datta D., Chaturvedi S.K., Nadarajan N., Varshney R.K. (2014). Genomics-assisted breeding in four major pulse crops of developing countries: Present status and prospects. Theor. Appl. Genet..

[54] Dadu R.H.R., Bar I., Ford R., Sambasivam P., Croser J., Ribalta F., Kaur S., Sudheesh S., Gupta D. (2021). *Lens orientalis* contributes quantitative trait loci and candidate genes associated with ascochyta blight resistance in lentil. Front. Plant Sci..

[55] Gupta N., Zargar S.M., Singh R., Nazir M., Mahajan R., Salgotra R. (2020). Marker association study of yield attributing traits in common bean (*Phaseolus vulgaris* L.). Mol. Biol. Rep..

[56] Mahajan R., Zargar S.M., Salgotra R.K., Singh R., Wani A.A., Nazir M., Sofi P.A. (2017). Linkage disequilibrium based association mapping of micronutrients in common bean (*Phaseolus vulgaris* L.): A collection of Jammu & Kashmir, India. 3 Biotech.

[57] Aguilar-Benitez D., Casimiro-Soriguer I., Maalouf F., Torres A.M. (2021). Linkage mapping and QTL analysis of flowering time in faba bean. Sci. Rep..

[58] Kahraman A., Demirel U., Ozden M., Muehlbauer F.J. (2010). Mapping of QTLs for leaf area and the association with winter hardiness in fall-sown lentil. Afr. J. Biotechnol..

[59] He Q., Yang H., Xiang S., Wang W., Xing G., Zhao T., Gai J. (2014). QTL mapping for the number of branches and pods using wild chromosome segment substitution lines in soybean [*Glycine max* (L.) Merr.]. Plant Genet. Resour..

[60] Abdi K., Deokar A., Banniza S., Tom W., Bunyamin T. (2016). QTL mapping of early flowering and resistance to ascochyta blight in chickpea (*Cicer arietinum* L.). Genome.

[61] Saxena R.K., Obala J., Sinjushin A., Kumar C.V.S., Saxena K.B., Varshney R.K. (2017). Characterization and mapping of *Dt1* locus which co-segregates with CcTFL1 for growth habit in pigeonpea. Theor. Appl. Genet..

[62] Breseghello F., Sorrels M.E. (2006). Association analysis as a strategy for improvement of qualitative traits in plants. Crop Sci..

[63] Mahajan R., Zargar S.M., Singh R., Salgotra R.K., Sufia F., Sonah H. (2017). Population structure analysis and selection of core set among common bean genotypes from Jammu and Kashmir, India. Appl. Biochem. Biotechnol..

[64] Huang C., Nie X., Shen C., You C., Li W., Zhao W., Zhang X., Lin Z. (2017). Population structure and genetic basis of the agronomic traits of upland cotton in China revealed by a genome-wide association study using high-density SNPs. Plant Biotechnol. J..

[65] Rostoks N.T., Ramsay L., Mackenzie K., Cardle L., Bhat P.R., Roose M.L., Svensson J.T., Stein N., Varshney R.K., Marshall D.F. (2006). Recent history of artificial outcrossing facilitates whole-genome association mapping in elite inbred crop varieties. Proc. Natl. Acad. Sci. USA.

[66] Binagwa P.H., Traore S.M., Egnin M., Bernard G.C., Ritte I., Mortley D., Kamfwa K., He G., Bonsi C. (2021). Genome-wide identification of powdery mildew resistance in common bean (*Phaseolus vulgaris* L.). Front Genet..

[67] Nabi A., Lateef I., Nisa Q., Banoo A., Rasool R.S., Shah M.D., Ahmad M., Padder B.A. (2022). *Phaseolus vulgaris-Colletotrichum lindemuthianum* pathosystem in the post-genomic era: An update. Curr. Microbiol..

[68] Perseguini J.M., Oblessuc P.R., Rosa J.R., Gomes K.A., Chiorato A.F., Carbonell S.A., Garcia A.A., Vianello R.P., Benchimol-Reis L.L. (2016). Genome-wide association studies of anthracnose and angular leaf spot resistance in common bean (*Phaseolus vulgaris* L.). PLoS ONE.

[69] Ambachew D., Blair M.W. (2021). Genome wide association mapping of root traits in the Andean genepool of common bean (*Phaseolus vulgaris* L.) grown with and without aluminum toxicity. Front. Plant Sci..

[70] Aldemir S., Ateş D., Temel H.Y., Yağmur B., Alsaleh A., Kahriman A., Hakan O., Albert V., Muhammed Bahattin T. (2017). QTLs for iron concentration in seeds of the cultivated lentil (*Lens culinaris* Medik.) via genotyping by sequencing. Turk. J. Agric. For..

[71] Chen Z., Ly V.J., Ly V.B., Buitink J., Leprince O., Verdier J. (2021). Genome-wide association studies of seed performance traits in response to heat stress in *Medicago truncatula* uncover miel1 as a regulator of seed germination plasticity. Front. Plant Sci..

[72] Kang Y., Sakiroglu M., Krom N., Stanton-Geddes J., Wang M., Lee Y.C., Young N.D., Udvardi M. (2015). Genome-wide association of drought-related and biomass traits with HapMap SNPs in *Medicago truncatula*. Plant Cell Environ..

[73] Kang Y., Torres-Jerez I., An Z., Greve V., Huhman D., Krom N., Udvardi M. (2019). Genome-wide association analysis of salinity responsive traits in *Medicago truncatula*. Plant Cell Environ..

[74] Sokolkova A., Burlyaeva M., Valiannikova T., Cui Y., Vishnyakova M., Schafleitner R., Lee C.R., Ting C.T., Nair R.M., Nuzhdin S. (2020). Genome-wide association study in accessions of the mini-core collection of mungbean (*Vigna radiata*) from the World Vegetable Gene Bank (Taiwan). BMC Plant Biol..

[75] Xu P., Wu X., Muñoz-Amatriaín M., Wang B., Wu X., Hu Y., Huynh B.L., Close T.J., Roberts P.A., Zhou W. (2017). Genomic regions, cellular components and gene regulatory basis underlying pod length variations in cowpea (*V. unguiculata* L. Walp). Plant Biotechnol. J..

[76] Hwang E.U., Song Q., Jia G., Specht J.E., Hyten D.L., Costa G.L., Cregan P.B. (2014). A genome wide association study of seed protein and oil content in soybean. BMC Genom..

[77] Raggi L., Caproni L., Carboni A., Negri V. (2019). Genome-wide association study reveals candidate genes for flowering time variation in common bean (*Phaseolus vulgaris* L.). Front. Plant Sci..

[78] Cichy K.A., Wiesinger J.A., Mendoza F.A. (2015). Genetic diversity and genome-wide association analysis of cooking time in dry bean (*Phaseolus vulgaris* L.). Theor. Appl. Genet..

[79] Yan L., Hofmann N., Li S., Ferreira M.E., Song B., Jiang G., Ren S., Quigley C., Fickus E., Cregan P. (2017). Identification of QTL with large effect on seed weight in a selective population of soybean with genome-wide association and fixation index analyses. BMC Genom..

[80] Rajendran K., Coyne C., Zheng P., Saha G., Main D., Amin N., Ma Y., Kisha T., Bett K.E., McGee R.J. (2021). Genetic diversity and GWAS of agronomic traits using an ICARDA lentil (*Lens culinaris* Medik.) reference plus collection. Plant Genet. Resour..

[81] Nkhata W., Shimelis H., Melis R., Chirwa R., Mzengeza T., Mathew I., Shayanowako A. (2021). Genome-wide association analysis of bean fly resistance and agro-morphological traits in common bean. PLoS ONE.

[82] Das S., Singh M., Srivastava R., Bajaj D., Saxena M.S., Rana J.C., Bansal K.C., Tyagi A.K., Parida S.K. (2016). MQTL-seq delineates functionally relevant candidate gene harbouring a major QTL regulating pod number in chickpea. DNA Res..

[83] Doddamani D., Katta M.A., Khan A.W., Agarwal G., Shah T.M., Varshney R.K. (2014). CicArMiSatDB: The chickpea microsatellite database. BMC Bioinformat..

[84] Doddamani D., Khan A.W., Katta M.A.V.S.K., Agarwal G., Thudi M., Ruperao P., Edwards D., Varshney R.K. (2015). CicArVarDB: SNP and InDel database for advancing genetics research and breeding applications in chickpea. Database.

[85] Gayali S., Acharya S., Lande N.V., Pandey A., Chakraborty S., Chakraborty N. (2016). CicerTransDB 1.0: A resource for expression and functional study of chickpea transcription factors. BMC Plant Biol..

[86] Sarika V.A., Iquebal M.A., Rai A., Kumar D. (2013). PIPEMicroDB: Microsatellite database and primer generation tool for pigeonpea genome. Database.

[87] Muranaka S., Shono M., Myoda T., Takeuchi J., Franco J., Nakazawa Y., Boukar O., Takaji H. (2016). Genetic diversity of physical, nutritional and functional properties of cowpea grain and relationships among the traits. Plant Genet. Resour..

[88] Grant D., Nelson R.T., Cannon S.B., Shoemaker R.C. (2010). SoyBase, the USDA-ARS soybean genetics and genomics database. Nucl. Acids Res..

[89] Pandey M.K., Agarwal G., Kale V., Clevenger J., Nayak S.N., Sriswathi M., Chitikineni A., Chavarro C., Chen X., Upadhyaya H.D. (2017). Development and evaluation of a high density genotyping ‘AxiomArachis’ array with 58K SNPs for accelerating genetics and breeding in groundnut. Sci. Rep..

[90] Roorkiwal M., Jain A., Kale S.M., Doddamani D., Chitikineni A., Thudi M., Varshney R.K. (2018). Development and evaluation of high-density Axiom^®^ Cicer SNP Array for high-resolution genetic mapping and breeding applications in chickpea. Plant Biotechnol. J..

[91] Munoz N., Liu A., Kan L., Li M.W., Lam H.M. (2017). Potential uses of wild germplasms of grain legumes for crop improvement. Int. J. Mol. Sci..

[92] Blair M.W., Cortés A.J., Farmer A.D., Huang W., Ambachew D., Penmetsa R.V., Carrasquilla-Garcia N., Assefa T., Cannon S.B. (2018). Uneven recombination rate and linkage disequilibrium across a reference SNP map for common bean (*Phaseolus vulgaris* L.). PLoS ONE.

[93] Song Q.L., Yan C., Quigley E., Fickus H., Wei L., Chen L., Dong F., Araya S., Liu J., Hyten D. (2020). Soybean BARCSoySNP6K: An assay for soybean genetics and breeding research. Plant J..

[94] Janila P., Nigam S.N., Pandey M.K., Nagesh P., Varshney R. (2013). Groundnut improvement: Use of genetic and genomic tools. Front. Plant Sci..

[95] Varshney R.K., Close T.J., Singh N.K., Hoisington D.A., Cook D.R. (2009). Orphan legume crops enter the genomics era!. Curr. Opin. Plant Biol..

[96] Guimaraes P.M., Garsmeur O., Proite K., Leal-Bertioli S.C., Seijo G., Chaine C., Bertioli D.J., D’Hont A. (2008). BAC libraries construction from the ancestral diploid genomes of the allotetraploid cultivated peanut. BMC Plant Biol..

[97] Kilian A. DArT-Based Whole Genome Profiling and Novel Information Technologies in Support System of Modern Breeding of Groundnut. Proceedings of the 3rd International Conference for Arachis through Genomics and Biotechnology (AAGB).

[98] Varshney R.K., Thudi M., May G.D., Jackson S.A. (2010). Legume genomics and breeding. Plant Breed. Rev..

[99] Knoll J.E., Ramos M.L., Zeng Y., Holbrook C.C., Chow M., Chen S., Maleki S., Bhattacharya A., Ozias-Akins P. (2011). TILLING for allergen reduction and improvement of quality traits in peanut (*Arachis hypogaea* L.). BMC Plant Biol..

[100] Arumuganathan K., Earle E.D. (1991). Nuclear DNA content of some important plant species. Plant Mol. Biol. Rep..

[101] Millan T., Winter P., Jungling R., Gil J., Rubio J., Cho S., Cobos M.J., Iruela M., Rajesh P.N., Tekeoglu M. (2010). A consensus genetic map of chickpea (*Cicer arietinum* L.) based on 10 mapping populations. Euphytica.

[102] Upadhyaya H.D., Thudi M., Dronavalli N., Gujaria N., Singh S., Sharma S., Varshney R.K. (2011). Genomic tools and germplasm diversity for chickpea improvement. Plant Genet. Resour..

[103] Varshney R.K., Hoisington D.A., Upadhyaya H.D., Gaur P.M., Nigam S.N., Saxena K., Vadez V., Sethy N.K., Bhatia S., Aruna R., Varshney R.K., Tuberosa R. (2007). Molecular Genetics and Breeding of Grain Legume Crops for the Semi-Arid Tropics. Genomics-Assisted Crop Improvement, 2. Genomics Applications in Crops.

[104] Jain A., Roorkiwal M., Kale S., Garg V., Yadala R., Varshney R.K. (2019). InDel markers: An extended marker resource for molecular breeding in chickpea. PLoS ONE.

[105] Varshney R.K., Kudapa H., Roorkiwal M., Mahendar T., Pandey M.K., Saxena R.K., Chamarthi S.K., Mohan S.M., Mallikarjuna N., Upadhyaya H. (2012). Advances in genetics and molecular breeding of three legume crops of semi-arid tropics using next-generation sequencing and high-throughput genotyping technologies. J. Biosci..

[106] Argout X., Salse J., Aury J.M., Guiltinan M.J., Droc G., Gouzy J., Allegre M., Chaparro C., Legavre T., Maximova S.N. (2011). The genome of *Theobroma cacao*. Nat. Genet..

[107] Yang S.Y., Saxena R.K., Kulwal P.A., Ash G.J., Dubey A., Harper D.I., Upadhyaya H.D., Gothalwal R., Kilian A., Varshney R.K. (2011). The first genetic map of pigeonpea based on diversity arrays technology (DArT) markers. J. Genet..

[108] Pedrosa-Harand A., Kami J., Gepts P., Geffroy V., Schweizer D. (2009). Cytogenetic mapping of common bean chromosomes reveals a less compartmentalized small-genome plant species. Chrom. Res..

[109] O’Rourke J.A., Iniguez L.P., Fu F., Bucciarelli B., Miller S.S., Jackson S.A., McClean P.E., Li J., Dai X., Zhao P.X. (2014). An RNA-Seq based gene expression atlas of the common bean. BMC Genom..

[110] Song Q., Jia G., Hyten D.L., Jenkins J., Hwang E.Y., Schroeder S.G., Osorno J.M., Schmutz J., Jackson S.A., McClean P.E. (2015). SNP assay development for linkage map construction, anchoring whole-genome sequence, and other genetic and genomic applications in common bean. G3 Genes|Genomes|Genet..

[111] Singh R.J., Hymowitz T. (1988). The genomic relationship between *Glycine max* L. Merr. and *G. soja* Sieb. and Zucc. as revealed by pachytene chromosome analysis. Theor. Appl. Genet..

[112] Song Q., Hyten D.L., Jia G., Quigley C.V., Fickus E.W., Nelson R.L., Cregan P.B. (2013). Development and evaluation of SoySNP50K, a high-density genotyping array for soybean. PLoS ONE.

[113] Jeong N., Kim K.S., Jeong S., Kim J.Y., Park S.K., Lee J.S., Jeong S.C., Kang S.T., Ha B.K., Kim D. (2019). Korean soybean core collection: Genotypic and phenotypic diversity population structure and genome-wide association study. PLoS ONE.

[114] Wang J., Chu S., Zhang H., Zhu Y., Cheng H., Yu D. (2016). Development and application of a novel genome-wide SNP array reveals domestication history in soybean. Sci. Rep..

[115] Kole C., Muthamilarasan M., Henry R., Edwards D., Sharma R., Abberton M., Batley J., Bentley A., Blakeney M., Bryant J. (2015). Application of genomics-assisted breeding for generation of climate resilient crops: Progress and prospects. Front. Plant Sci..

[116] Chandra A.K., Kumar A., Bharati A., Joshi R., Agrawal A., Kumar S. (2020). Microbial-assisted and genomic-assisted breeding: A two-way approach for the improvement of nutritional quality traits in agricultural crops. 3 Biotech.

[117] Dikshit H., Singh A., Singh D., Aski M., Jain N., Hegde V.S., Basandrai A., Basandrai D., Sharma T. (2016). Tagging and mapping of SSR marker for rust resistance gene in lentil (*Lens culinaris* Medikus subsp. *culinaris*). Indian J. Exp. Biol..

[118] Polanco C., Saenz de Miera L.E., Gonzalez A.I., Garcia P., Fratini R., Vaquero F., Vences F.J., Pérez de la Vega M. (2019). Construction of a highdensity interspecific (*Lens culinaris* x *L. odemensis*) genetic map based on functional markers for mapping morphological and agronomical traits, and QTLs affecting resistance to ascochyta in lentil. PLoS ONE.

[119] Singh G., Singh I., Taggar G.K., Rani U., Sharma P., Gupta M., Singh S. (2020). Introgression of productivity enhancing traits, resistance to pod borer and *Phytopthora* stem blight from *Cajanus scarabaeoides* to cultivated pigeonpea. Physiol. Mol. Biol. Plants.

[120] Wu L., Fredua-Agyeman R., Hwang S.F., Chang K.F., Conner R.L., McLaren D.L., Strelkov S.E. (2021). Mapping QTL associated with partial resistance to Aphanomyces root rot in pea (*Pisum sativum* L.) using a 13.2 K SNP array and SSR markers. Theor. Appl. Genet..

[121] Meuwissen T. (2007). Genomic selection: Marker assisted selection on a genome wide scale. J. Anim. Breed. Genet..

[122] Goddard M.E., Hayes B.J. (2007). Genomic selection. J. Anim. Breed. Genet..

[123] Xu Y., Liu X., Fu J., Wang H., Wang J., Huang C., Prasanna B.M., Olsen M.S., Wang G., Zhang A. (2020). Enhancing genetic gain through genomic selection: From livestock to plants. Plant Comm..

[124] Goddard M. (2009). Genomic selection: Prediction of accuracy and maximisation of long term response. Genetica.

[125] Crossa J., Perez-Rodriguez P., Cuevas J., Montesinos-Lopez O., Jarquin D., de los Campos G., Burgueño J., Juan M., Camacho G., Pérez-Elizalde S. (2017). Genomic selection in plant breeding: Methods, models, and perspectives. Trends Plant Sci..

[126] Langridge P., Reynolds M.P. (2015). Genomic tools to assist breeding for drought tolerance. Curr. Opin. Biotechnol..

[127] Wang X., Xu Y., Hu Z., Xu C. (2008). Genomic selection methods for crop improvement: Current status and prospects. Crop J..

[128] Jain A., Roorkiwal M., Pandey M.K., Varshney R.K. (2017). Current Status and Prospects of Genomic Selection in Legumes. Genomic Selection for Crop Improvement: New Molecular Breeding Strategies for Crop. Improvement.

[129] Batieno B.J., Danquah E., Tignegre J.B., Huynh B.L., Drabo I., Close T., Ofori K., Roberts P., Ouedraogo T.J. (2016). Application of marker-assisted backcrossing to improve cowpea (*Vigna unguiculata* L. Walp) for drought tolerance. J. Plant. Breed. Crop Sci..

[130] Schneider K.A., Rosales-Serna R., Ibarra-Perez F., Cazares-Enriquez B., Acosta-Gallegos J.A., Ramirez-Vallejo P., Wassimi N., Kelly J.D. (1997). Improving common bean performance under drought stress. Crop Sci..

[131] Zargar S.M., Nazir M., Gupta M., Farhat S., Mahajan R., Salgotra R.K., Mir R.A. (2014). Molecular marker assisted approaches (MMAA) for enhancing low water stress tolerance in common bean: An update. Mol. Plant Breed..

[132] Diaz M.L., Ricaurte J., Tovar E., Cajiao C.E., Teran H., Grajales M., Polanıa J., Rao I., Beebe S., Raatz B. (2018). QTL analyses for tolerance to abiotic stresses in a common bean (*Phaseolus vulgaris* L.) population. PLoS ONE.

[133] Vasconcellos R.C., Oraguzie O.B., Soler A., Arkwazee H., Myers J.R., Ferreira J.J., Song Q., McClean P., Miklas P.N. (2017). Meta-QTL for resistance to white mold in common bean. PLoS ONE.

[134] Garzon L.N., Ligarreto G.A., Blair M.W. (2008). Molecular marker-assisted backcrossing of anthracnose resistance into andean climbing beans (*Phaseolus vulgaris* L.). Crop Sci..

[135] Blair M.W., Muñoz C., Buendía H.F., Flower J., Bueno J.M., Cardona C. (2010). Genetic mapping of microsatellite markers around the arcelin bruchid resistance locus in common bean. Theor. Appl. Genet..

[136] Singh D., Singh C.K., Tomar R.S.S., Sharma S., Karwa S., Pal M., Singh V., Sanwal S.K., Sharma P.C. (2020). Genetics and molecular mapping for salinity stress tolerance at seedling stage in lentil (*Lens culinaris* Medik). Crop Sci..

[137] Tayeh N., Aluome C., Falque M., Jacquin F., Klein A., Chauveau A., Bérard A., Houtin H., Rond A., Kreplak J. (2015). Development of two major resources for pea genomics: The GenoPea 13.2K SNP Array and a high-density, high-resolution consensus genetic map. Plant J..

[138] Katoch V., Sharma S., Pathania S., Banayal D., Sharma S., Rathour R. (2010). Molecular mapping of pea powdery mildew resistance gene *er2* to pea linkage group III. Mol. Breed..

[139] Cobos M.J., Satovic Z., Rubiales D., Fondevilla S. (2018). *Er3* gene, conferring resistance to powdery mildew in pea, is located in pea LGIV. Euphytica.

[140] Leonforte A., Forster J., Redden R., Nicolas M., Salisbury P. (2013). Sources of high tolerance to salinity in pea (*Pisum sativum* L.). Euphytica.

[141] Kim M., Hyten D.L., Niblack T.L., Diers B.W. (2011). Stacking resistance alleles from wild and domestic soybean sources improves soybean cyst nematode resistance. Crop Sci..

[142] Arahana V.S., Graef G.L., Specht J.E., Steadman J.R., Eskridge K.M. (2001). Identification of QTLs for resistance to in soybean. Crop Sci..

[143] Bachman M.S., Tamulonis J.P., Nickell C.D., Bent A.F. (2001). Molecular markers linked to brown stem rot resistance genes, *Rbs1* and *Rbs2*, in soybean. Crop Sci..

[144] Yang W., Weaver D., Nielsen B., Qiu J., Salamini F. (2001). Molecular mapping of a new gene for resistance to frogeye leaf spot of soya bean in ‘Peking’. Plant Breed..

[145] Ramalingam J., Alagarasan G., Savitha P., Lydia K., Pothiraj G., Vijayakumar E., Sudhagar R., Singh A., Vedna K., Vanniarajan C. (2020). Improved host-plant resistance to *Phytophthora* rot and powdery mildew in soybean (*Glycine max* (L.) Merr.). Sci. Rep..

[146] Wang D.G., Lin Z., Kai L., Ying M., Wang L.Q., Yang Y.Q., Yang Y.H., Zhi H.J. (2017). Marker-assisted pyramiding of soybean resistance genes *RSC4, RSC8*, and *RSC14Q* to soybean mosaic virus. J. Integr. Agric..

[147] Kumar V., Rani A., Rawal R. (2013). Deployment of gene specific marker in development of kunitz trypsin inhibitor free soybean genotypes. Indian J. Exp. Biol..

[148] Kumar V., Rani A., Rawal R., Mourya V. (2015). Marker assisted accelerated introgression of null allele of kunitz trypsin inhibitor in soybean. Breed. Sci..

[149] Maranna S., Verma K., Talukdar A., Lal S.K., Kumar A., Mukherjee K. (2016). Introgression of null allele of kunitz trypsin inhibitor through marker-assisted backcross breeding in soybean (*Glycine max* L. Merr.). BMC Genet..

[150] Rawal R., Kumar V., Rani A., Gokhale S.M. (2020). Genetic elimination of off-flavour generating lipoxygenase-2 gene of soybean through marker assisted backcrossing and its effect on seed longevity. Plant Breed. Biotech..

[151] Varshney R.K., Pandey M.K., Janila P., Nigam S.N., Sudini H., Gowda M.V., Sriswathi M., Radhakrishnan T., Manohar S.S., Nagesh P. (2014). Marker-assisted introgression of a QTL region to improve rust resistance in three elite and popular varieties of peanut (*Arachis hypogaea* L.). Theor. Appl. Genet..

[152] Chu Y., Gill R., Clevenger J., Timper P., Holbrook C.C., Ozias-Akins P. (2016). Identification of rare recombinants leads to tightly linked markers for nematode resistance in peanut. Peanut Sci..

[153] Chu Y., Wu C.L., Holbrook C.C., Tillman B.L., Person G., Ozias-Akins P. (2011). Marker-assisted selection to pyramid nematode resistance and the high oleic trait in peanut. Plant Genome.

[154] Janila P., Pandey M.K., Manohar S.S., Variath M.T., Nallathambi P., Nadaf H.L., Sudini H., Varshney R.K. (2016). Foliar fungal disease-resistant introgression lines of groundnut (*Arachis hypogaea* L.) record higher pod and haulm yield in multilocation testing. Plant Breed..

[155] Shasidhar Y., Variath M.T., Vishwakarma M.K., Manohar S.S., Gangurde S.S., Sriswathi M., Sudini H.K., Dobariya K.L., Bera S.K., Radhakrishnan T. (2020). Improvement of three Indian popular groundnut varieties for foliar disease resistance and high oleic acid using SSR markers and SNP array in marker-assisted backcrossing. Crop. J..

[156] Varshney R.K., Mohan S.M., Gaur P.M., Chamarthi S.K., Singh V.K., Srinivasan S., Swapna N., Sharma M., Pande S., Singh S. (2014). Marker-assisted backcrossing to introgress resistance to fusarium wilt race 1 and ascochyta blight in C 214, an elite cultivar of chickpea. Plant Genome.

[157] Boukar O., Fatokun C.A., Huynh B.L., Roberts P.A., Close T.J. (2016). Genomic tools in cowpea breeding programs: Status and perspectives. Front. Plant Sci..

[158] Souleymane A., Aken’Ova M., Fatokun C., Alabi O. (2013). Screening for resistance to cowpea aphid (*Aphis craccivora* koch) in wild and cultivated cowpea (*Vigna unguiculata* L. Walp.) accessions. Int. J. Sci. Environ. Technol..

[159] Kongjaimun A., Kaga A., Tomooka N., Somta P., Shimizu T., Shu Y., Isemura T., Vaughan D.A., Srinives P. (2012). An SSR-based linkage map of yardlong bean (*Vigna unguiculata* (L.) Walp. subsp. *unguiculata* Sesquipedalis group) and QTL analysis of pod length. Genome.

[160] Kongjaimun A., Somta P., Tomooka N., Kaga A., Vaughan D.A., Srinives P. (2013). QTL mapping of pod tenderness and total soluble solid in yardlong bean [*Vigna unguiculata* (L.) Walp. subsp. *unguiculata* cv.-gr. *sesquipedalis*]. Euphytica.

[161] Andargie M., Knudsen J.T., Pasquet R.S., Gowda B.S., Muluvi G.M., Timko M.P. (2014). Mapping of quantitative trait loci for floral scent compounds in cowpea (*Vigna unguiculata* L.). Plant Breed..

[162] Ravelombola W., Shi A., Huynh B.L. (2021). Loci discovery, network-guided approach, and genomic prediction for drought tolerance index in a multi-parent advanced generation intercross (MAGIC) cowpea population. Hort. Res..

[163] Paudel D., Dareus R., Rosenwald J., Muñoz-Amatriaín M., Rios E.F. (2021). Genome-wide association study reveals candidate genes for flowering time in cowpea (*Vigna unguiculata* [L.] Walp.). Front. Genet..

[164] Wu X., Cortés A.J., Blair M.W. (2022). Genetic differentiation of grain, fodder and pod vegetable type cowpeas (*Vigna unguiculata* L.) identified through single nucleotide polymorphisms from genotyping-by-sequencing. Mol. Hort..

[165] Zhang D., Cheng H., Wang H., Zhang H.Y., Liu C.Y., Yu D.Y. (2010). Identification of genomic regions determining flower and pod numbers development in soybean (*Glycine max* L.). J. Genet. Genom..

[166] Kerem Z., Lev-Yadun S., Gopher A., Weinberg P., Abbo S. (2010). Chickpea domestication in the neolithic levant through the nutritional perspective. J. Archaeol. Sci..

[167] Warkentin T., Banniza S., Vandenberg A. (2005). CDC frontier kabuli chickpea. Can. J. Plant Sci..

[168] Moreno M.T., Cubero J. (1978). Variation in *Cicer arietinum* L.. Euphytica.

[169] Jain M., Misra G., Patel R.K., Priya P., Jhanwar S., Khan A.W., Shah N., Singh V.K., Garg R., Jeena G. (2013). A draft genome sequence of the pulse crop chickpea (*Cicer arietinum* L.). Plant J..

[170] Bajaj D., Das S., Upadhyaya H.D., Ranjan R., Badoni S., Kumar V., Tripathi S., Gowda C.L., Sharma S., Singh S. (2015). A genome-wide combinatorial strategy dissects complex genetic architecture of seed coat color in chickpec. Front. Plant Sci..

[171] Upadhyaya H.D., Bajaj D., Das S., Saxena M.S., Badoni S., Kumar V., Tripathi S., Gowda C., Sharma S., Tyagi A.K. (2015). A genome-scale integrated approach aids in genetic dissection of complex flowering time trait in chickpea. Plant Mol. Biol..

[172] Srivastava R., Singh M., Bajaj D., Parida S.K. (2016). A high-resolution *InDel* (insertion–deletion) markers-anchored consensus genetic map identifies major QTLs governing pod number and seed yield in chickpea. Front. Plant Sci..

[173] Gupta C., Gupta M., Gupta S., Salgotra R.K. (2022). Screening of common bean (*Phaseolus vulgaris* L.) germplasm against *Colletotrichum lindemuthianum* inciting bean anthracnose. Res. J. Biotech..

[174] Assefa E. (2020). Application of biotechnological tools for common bean (*Phaseolus vulgaris* L.) improvement. Zenodo.

[175] Vlasova A., Capella-Gutiérrez S., Rendón-Anaya M., Hernández-Oñate M., Minoche A.E., Erb I., Camara F., Prieto-Barja P., Corvelo A., Sanseverino W. (2016). Genome and transcriptome analysis of the Mesoamerican common bean and the role of gene duplications in establishing tissue and temporal specialization of genes. Genome Biol..

[176] Thudi M., Palakurthi R., Schnable J.C., Chitikineni A., Dreisigacker S., Mace E., Srivastava R.K., Satyavathi C.T., Odeny D., Tiwari V.K. (2021). Genomic resources in plant breeding for sustainable agriculture. J. Plant Physiol..

[177] Blair M.W., Astudillo C., Rengifo J., Beebe S.E., Graham R. (2011). QTL analyses for seed iron and zinc concentrations in an intra-genepool population of andean common beans (*Phaseolus vulgaris* L.). Theor. Appl. Genet..

[178] Miklas P.N., Kelly J.D., Singh S.P. (2003). Registration of anthracnose resistant pinto bean germplasm line USPT-ANT-1. Crop Sci..

[179] Miklas P.N., Kelly J.D., Beebe S.E., Blair M.W. (2006). Common bean breeding for resistance against biotic and abiotic stresses: From classical to MAS breeding. Euphytica.

[180] Obala J., Mukankusi C., Rubaihayo P.R., Gibson P., Edema R. (2012). Improvement of resistance to Fusarium root rot through gene pyramiding in common bean. Afr. Crop Sci. J..

[181] Nzungize J., Gepts P., Buruchara R.A., Male A., Ragama P., Busogoro J.P., Baudoin J.P. (2011). Introgression of pythium root rot resistance gene into Rwandan susceptible common bean cultivars. Afr. J. Plant Sci..

[182] Uwera A., Rusagara J., Msolla S., Musoni A., Assefa T. (2021). Molecular marker-assisted backcrossing of anthracnose resistance genes into common beans (*Phaseolus vulgaris* L.) varieties. Am. J. Plant Sci..

[183] Keller B., Ariza-Suarez D., de la Hoz J., Aparicio J.S., Portilla-Benavides A.E., Buendia H.F., Mayor V.M., Studer B., Raatz B. (2020). Genomic prediction of agronomic traits in common bean (*Phaseolus vulgaris* L.) under environmental stress. Front. Plant Sci..

[184] Pandey M.K., Pandey A.K., Kumar R., Nwosu C.V., Guo B., Wright G.C., Bhat R.S., Chen X., Bera S.K., Yuan M. (2020). Translational genomics for achieving higher genetic gains in groundnut. Theor. Appl. Genet..

[185] Akohoue F., Achigan-Dako E.G., Sneller C., Van Deynze A., and Sibiya J. (2020). Genetic diversity, SNP-trait associations and genomic selection accuracy in a west African collection of Kersting’s groundnut [*Macrotyloma geocarpum*(Harms) Maréchal & Baudet]. PLoS ONE.

[186] Pazhamala L., Saxena R.K., Singh V.K., Sameerkumar C.V., Kumar V., Sinha P., Patel K., Obala J., Kaoneka S.R., Tongoona P. (2015). Genomics-assisted breeding for boosting crop improvement in pigeonpea (*Cajanus cajan*). Front. Plant Sci..

[187] Saxena R.K., Hake A., Hingane A.J., Kumar C.V.S., Bohra A., Sonnappa M., Rathore A., Kumar A.V., Mishra A., Tikle A.N. (2020). Translational pigeonpea genomics consortium for accelerating genetic gains in pigeonpea (*Cajanus cajan* L.). Agronomy.

[188] Bohra A., Mir R.R., Jha R., Maurya A.K., Varshney R.K., Narendra T., Renu T., Nishat P., Shabnam K.S. (2020). Advances in Genomics and Molecular Breeding for Legume Improvement. Advancement in Crop Improvement Techniques.

[189] Tullu A., Bett K., Banniza S., Vail S., Vandenberg A. (2013). Widening the genetic base of cultivated lentil through hybridization of *Lens culinaris* “Eston” and *L. ervoides* accession IG72815. Can. J. Plant Sci..

[190] Matthew R.S., Jennifer D., Muhammad J., Shimna S., Sara B., John F.W., Kaur S. (2017). Molecular breeding for ascochyta blight resistance in lentil: Current progress and future directions. Front. Plant Sci..

[191] Sharpe A.G., Ramsay L., Sanderson L.A., Fedoruk M.J., Clarke W.E., Li R., Kagale S., Vijayan P., Vandenberg A., Bett K.E. (2013). Ancient orphan crop joins modern era: Gene-based SNP discovery and mapping in lentil. BMC Genom..

[192] Duran Y., Fratini R., Garcia P., Pérez de la Vega M. (2004). An intersubspecific genetic map of Lens. Theor. Appl. Genet..

[193] Hamwieh A., Udupa S., Choumane W., Sarker A., Dreyer F., Jung C., Baum M. (2005). A genetic linkage map of *Lens* sp. based on microsatellite and AFLP markers and the localization of fusarium vascular wilt resistance. Theor. Appl. Genet..

[194] Aldemir S.B., Sever T., Ates D., Yagmur B., Kaya H.B., Temel H.Y., Kahriman A., Ozkan H., Tanyolac M.B. QTL Mapping of Genes Controlling Fe Uptake in Lentil (*Lens culinaris* L.) Seed Using Recombinant Inbred Lines. Proceedings of the Plant and Animal Genome Conference XXII P3360.

[195] Boutet G., Alves Carvalho S., Falque M., Peterlongo P., Lhuillier E., Bouchez O., Lavaud C., Pilet-Nayel M.L., Riviere N., Baranger A. (2016). SNP discovery and genetic mapping using genotyping by sequencing of whole genome genomic DNA from a pea RIL population. BMC Genom..

[196] Haggard J.E. (2007). Characterization of physiological resistance to white mold and search for molecular markers linked to resistance via advanced backcross QTL analysis in an interspecific cross between *Phaseolus coccineus* and *P. vulgaris*. HortSci.

[197] Fondevilla S., Satovic Z., Rubiales D., Moreno M.T., Torres A.M. (2008). Mapping of quantitative trait loci for resistance to *Mycosphaerella pinodes* in *Pisum sativum* subsp. *syriacum*. Mol. Breed..

[198] Devi J., Mishra G.P., Sagar V., Kaswan V., Dubey R.K., Singh P.M., Sharma S.K., Behera T.K. (2022). Gene-based resistance to *Erysiphe* species causing powdery mildew disease in peas (*Pisum sativum* L.). Genes.

[199] Annicchiarico P., Nazzicari N., Pecetti L., Romani M., Russi L. (2019). Pea genomic selection for Italian environments. BMC Genom..

[200] Baldermann S., Blagojevic L., Frede K., Klopsch R., Neugart S., Neumann A., Ngwene B., Norkeweit J., Schröter D., Schröter A. (2016). Are neglected plants the food for the future?. Crit. Rev. Plant Sci..

[201] Popoola J., Ojuederie O., Omonhinmin C., Adegbite A., Shah F., Khan Z., Iqbal A., Turan M., Olgun M. (2019). Neglected and underutilized legume crops: Improvement and future prospects. Recent Advances in Grain Crops Research.

[202] Rathi D., Chakraborty S., Chakraborty N. (2021). Grasspea, a critical recruit among neglected and underutilized legumes, for tapping genomic resources. Curr. Plant Biol..

[203] Plewinski P., Książkiewicz M., Rychel-Bielska S., Rudy E., Wolko B. (2019). Candidate domestication-related genes revealed by expression quantitative trait loci mapping of narrow-leafed lupin (*Lupinus angustifolius* L.). Int. J. Mol. Sci..

[204] Reddy V.R., Das S., Dikshit H.K., Mishra G.P., Aski M.S., Singh A., Tripathi K., Pandey R., Bansal R., Pal Singh M. (2021). Genetic dissection of phosphorous uptake and utilization efficiency traits using GWAS in mungbean. Agronomy.

[205] Rychel S., Książkiewicz M. (2019). Development of gene-based molecular markers tagging low alkaloid pauper locus in white lupin (*Lupinus albus* L.). J. Appl. Genet..

[206] Somta P., Jomsangawong A., Yundaeng C., Yuan X., Chen J., Tomooka N., Chen X. (2018). Genetic dissection of Azuki bean weevil (*Callosobruchus chinensis* L.) resistance in moth bean (*Vigna aconitifolia* [Jaqc.] Maréchal). Genes.

